# Increased Krüppel-like factor 12 impairs embryo attachment via downregulation of leukemia inhibitory factor in women with recurrent implantation failure

**DOI:** 10.1038/s41420-018-0088-8

**Published:** 2018-08-06

**Authors:** Chenyang Huang, Haixiang Sun, Zhilong Wang, Yang Liu, Xi Cheng, Jingyu Liu, Ruiwei Jiang, Xindong Zhang, Xin Zhen, Jidong Zhou, Linjun Chen, Lijun Ding, Guijun Yan, Yue Jiang

**Affiliations:** Reproductive Medicine Center, The Affiliated Drum Tower Hospital of Nanjing University Medical School, Nanjing, 210008 China

**Keywords:** Infertility, Transcriptional regulatory elements

## Abstract

Recurrent implantation failure (RIF) caused by various etiological factors remains a challenge for fertility clinicians using assisted reproductive technology (ART) worldwide. Dysregulation of leukemia inhibitory factor (LIF) in the endometria of women with RIF is involved in impaired endometrial receptivity and embryo adhesion. However, the mechanism through which LIF expression is regulated in women with RIF is still poorly understood. Our previous study noted that the abnormally increased endometrial Krüppel-like factor 12 (KLF12) in RIF women led to impaired decidualization and embryo implantation. Here, we further found that KLF12 inhibited embryo adhesion in vivo and in vitro by repressing LIF expression. Mechanistically, KLF12 bound to conserved sites (CAGTGGG, −6771 to −6765 and −7115 to −7109) within the LIF promoter region and repressed LIF transcription directly. Exogenous LIF significantly reversed the KLF12-mediated repression of BeWo spheroid adhesion. KLF12 expression was reduced significantly in Ishikawa cells treated with progestogen, which was due to the activation of Akt signaling. These findings may provide novel potential therapeutic regimens for patients with RIF and disrupted endometrial receptivity.

## Introduction

Despite significant improvements in assisted reproductive technology (ART), a number of patients experience ART failure after repeated attempts^1^. The failure to achieve pregnancy after at least four good-quality embryo transfers over a minimum of three fresh or frozen in vitro fertilization (IVF) cycles is defined as recurrent implantation failure (RIF)^2–4^. RIF still remains a major challenge for both clinicians and researchers toward improving pregnancy outcomes in ART.

Embryo implantation involves a complex series of events that results in the establishment of a connection between maternal and embryonic sections^5,6^. A receptive endometrium together with a good-quality embryo and an efficient maternal embryo signaling process are necessary for successful implantation to occur^6,7^. Impaired endometrial receptivity has become a significant limiting factor for embryo attachment, as the quality of embryos during IVF cycles has improved in recent years^8^. Leukemia inhibitory factor (LIF), an IL-6-family cytokine, is regarded as an important local mediator in the endometrium for successful implantation^9,10^. Increasing evidence indicates that LIF plays a critical role in the interaction between the embryo and the receptive endometrium^11^, as proved by LIF knockout mouse research, which confirmed that LIF was necessary for embryo attachment to the endometrial luminal epithelium^12^. LIF plays important roles in the complex events of successful pregnancy by binding with LIF receptor (LIFR) and activating the signal transducers and activators of transcription 3 (STAT3) signaling pathway^13–16^. There is increasing evidence that LIF-mediated STAT3 activation regulates endometrial receptivity^14,17^. Decreased LIF secretion has been described in a number of diseases, including adenomyosis^18^, genital tuberculosis^19^, and implantation failure^20^. Although LIF is essential for embryo-endometrial adhesion, there is limited research regarding the regulatory mechanism of LIF expression.

Several associated transcription factors reportedly participate in the preparation of human endometrial receptivity, such as homeobox containing transcription factor 10 (Hoxa10)^21^, signal transducer and activator of transcription 3 (STAT3)^22^, and activating transcription factor 3 (ATF3)^23^. Furthermore, LIF is considered to be a direct downstream target gene of ATF3^23^. We have previously demonstrated that Krüppel-like factor 12 (KLF12) is expressed in the glandular epithelium (GE) and stromal cells of secretory-phase endometrial tissue and that KLF12 negatively regulates endometrial decidualization through the transcriptional repression of target genes both in vitro and in vivo^24^.

This study reveals the excessive expression of KLF12 and decreased expression of LIF in the endometria of RIF patients. KLF12 is shown to be a novel downstream target gene that is reduced by medroxyprogesterone acetate (MPA) in Ishikawa cells, and LIF is considered to be a factor that is induced factor by estrogen (17β-estradiol, E_2_) and MPA. Because LIF can be transcriptionally regulated by other factors, to elucidate the exact mechanism of the interaction between KLF12 and LIF, we visually scanned the wild-type LIF sequence (−8000 to +100 relative to the transcriptional start site) and found two specific KLF12-binding sites in the promoter. Therefore, we hypothesize that in patients with RIF, enhanced KLF12 expression impairs endometrial receptivity by transcriptionally repressing LIF. These results offer a new molecular mechanism to consider in studies of the capacity of endometrial epithelial cells during embryo implantation.

## Results

### Increased expression of KLF12 in the endometria of patients with RIF

**Fig. 1:**
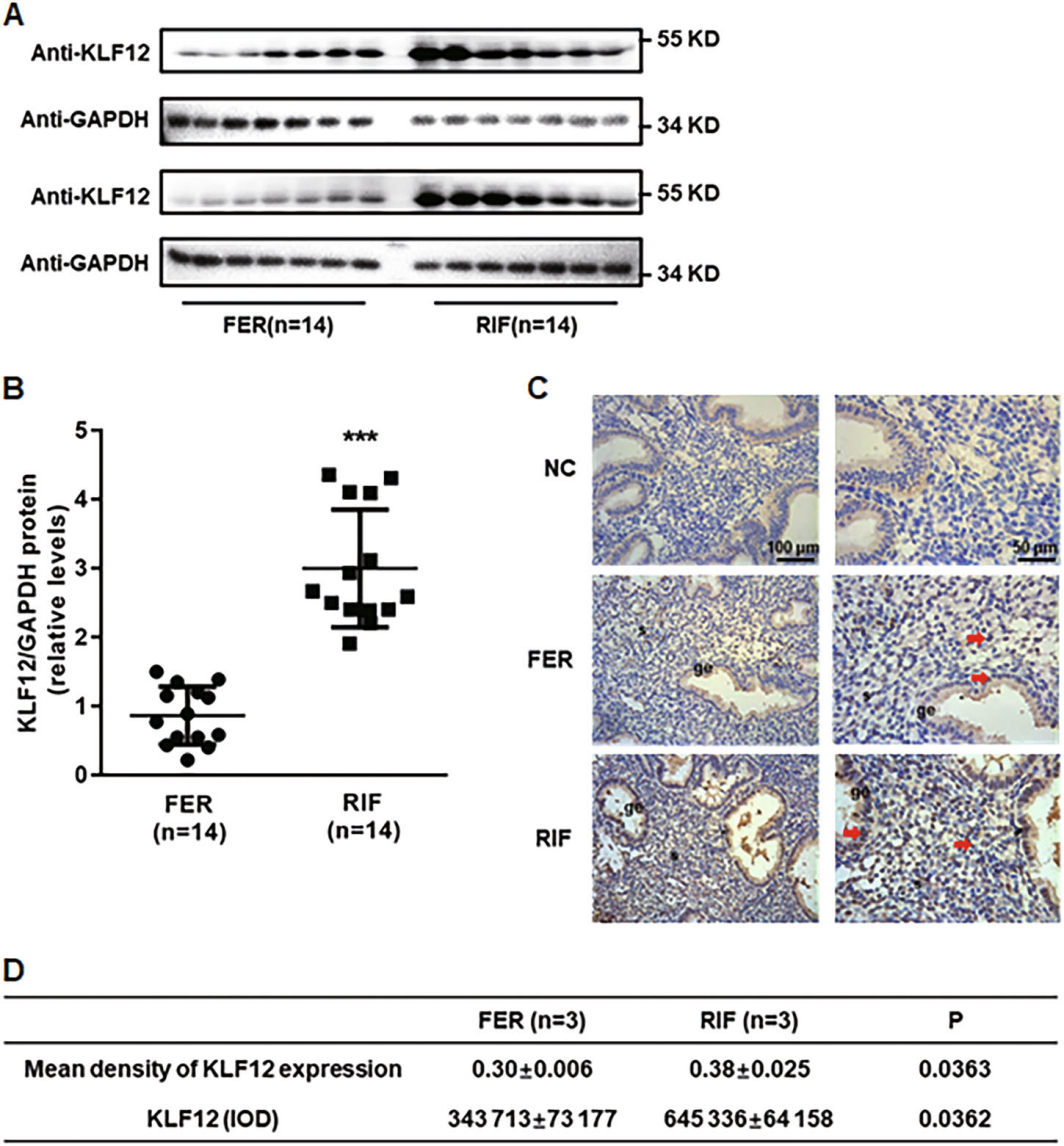
Aberrant expression of KLF12 in the endometria of RIF patients. **a**, **b** The total KLF12 protein levels were normalized to GAPDH expression, and the data for all of the endometrial samples are shown in the scatter plots. ^***^P < 0.001 compared with the FER group. **c**, **d** Immunohistochemical analysis was performed using KLF12 antibody. Endometrial tissue samples from fertile women and RIF patients are shown at ×200 (left panel) and ×400 (right panel) magnification. The negative control (NC) is nonspecific rabbit serum. Red represents positive staining (arrows). Scale bars, 100 μm (left panel) and 50 μm (right panel). The means and integrated optical densities of KLF12 expression in the endometrial tissue were calculated

The endometrial KLF12 protein levels were markedly increased (by greater than 3-fold) during the implantation window in the RIF patients compared with the control women (Fig. 1a, b). Furthermore, as demonstrated in immunolocalization analysis, the KLF12 protein abundance was greater in the endometria of the women with RIF than in the endometria of the fertile controls, especially in the endometrial epithelial cells in the RIF tissue samples (Fig. 1c, d), which indicated that KLF12 might play critical roles in embryo adhesion to the endometrial epithelial cells.

### KLF12 impairs embryo attachment

**Fig. 2:**
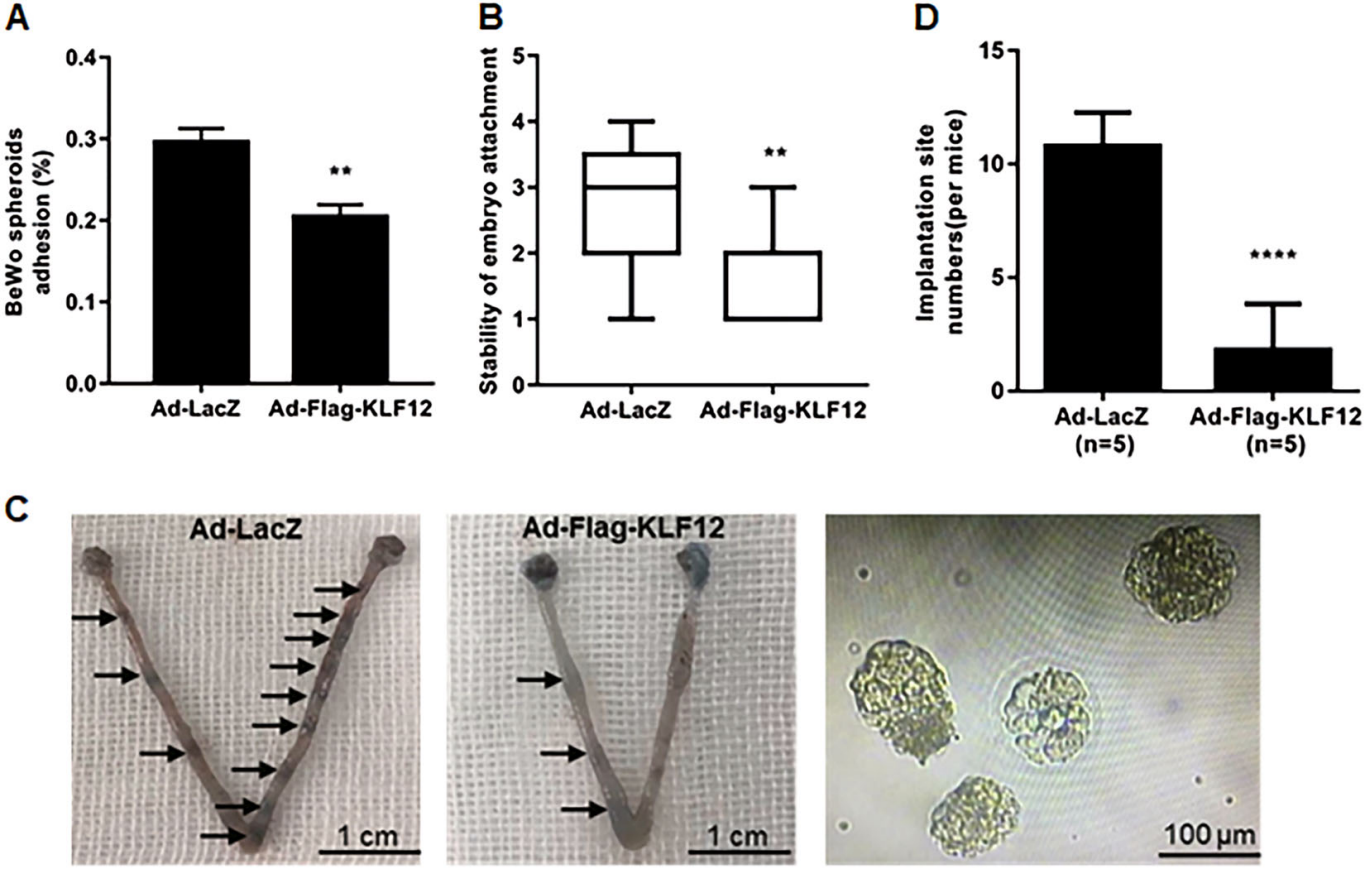
KLF12 inhibits embryo implantation. **a** Adhesion experiments with BeWo spheroids attached to the Ishikawa cell monolayer with indicated treatments. The data are the average of three independent experiments. ^**^P < 0.01 compared with the control group. **b** Adhesion experiments with mouse embryos attached to the Ishikawa cell monolayer with indicated treatments. The results are expressed as the median with the interquartile range of three independent experiments; boxes indicate quartiles, and whiskers indicate the range. ^**^P < 0.01 compared with the control group. **c** Uteri from KLF12-enhanced and LacZ mice at 4.5 dpc were acquired and stained with Chicago blue dye. The uteri were flushed, and the non-implanted blastocysts were then counted. Representative images are shown. **d** The number of implantation sites was counted. n is shown in each column. ^***^P < 0.001 compared with the control group

An appropriate trophoblast-epithelial cell interaction model confirmed that Ishikawa cell infection with Ad-Flag-KLF12 resulted in a significant reduction in BeWo spheroid adhesion (Fig. 2a). Next, an in vitro model of mouse blastocyst attachment showed that embryos incubated with KLF12-enhanced cells had lower attachment scores, with a median of 2 (1–3), than Ad-LacZ-treated cells, with a median score of 3 (2–4) (Fig. 2b). The uterine KLF12-overexpressing mice had fewer implantation sites on 4.5 days post-coitus (dpc) than the control group (KLF12 1.8 ± 0.9165 vs LacZ 10.8 ± 0.6633, *p* < 0.001) (Fig. 2c, d), further showing that KLF12 had an impact on embryo adhesion. In addition, the average number of non-implanted blastocysts in the KLF12-overexpressing uteri was 3.33 ± 0.6667, whereas we did not observe any non-implanted blastocysts in the control group.

### KLF12 is a downstream gene that is regulated by progesterone

**Fig. 3:**
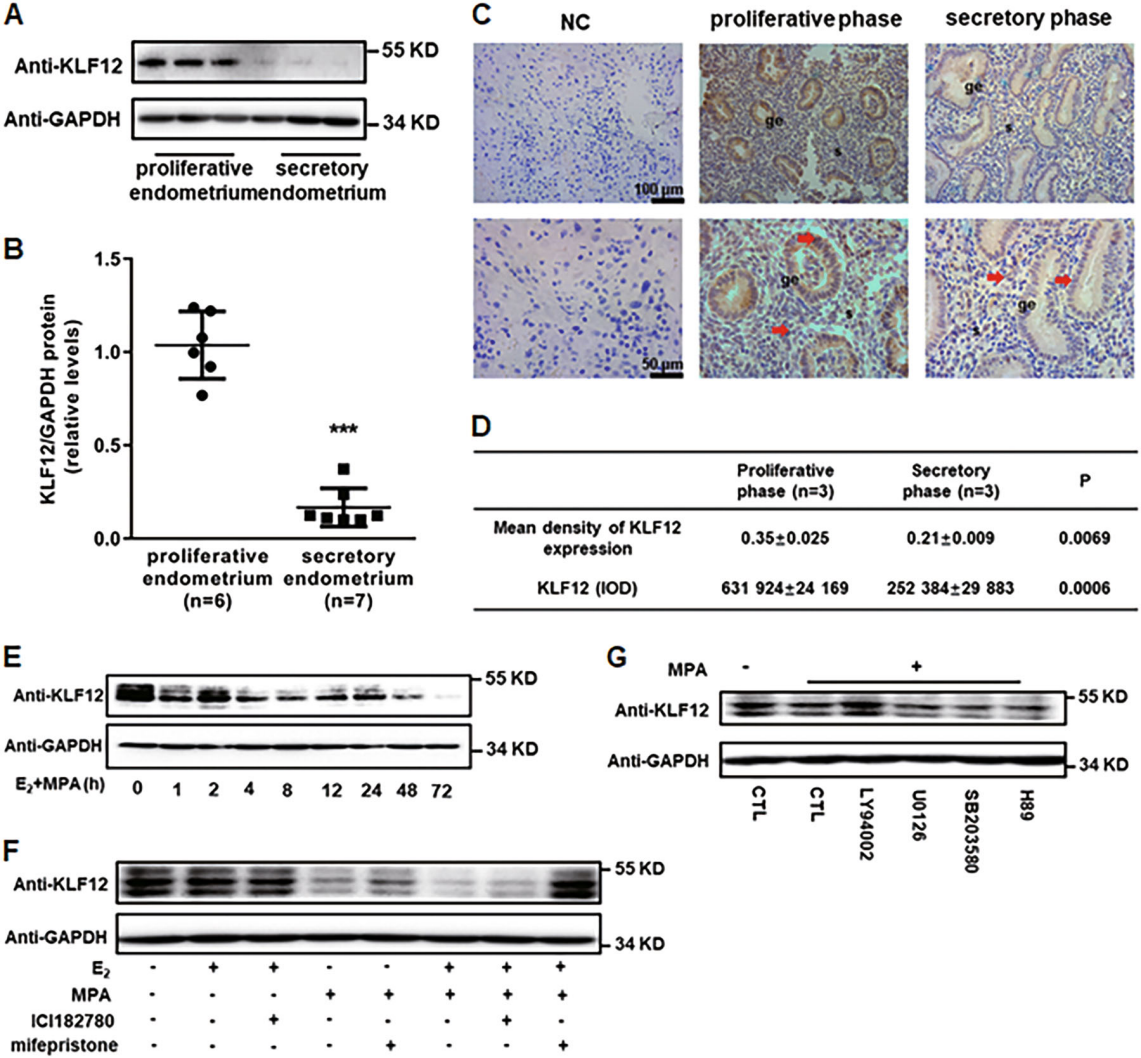
KLF12 is a downstream gene that is regulated by progesterone. **a**, **b** The total KLF12 protein levels of proliferative- and secretory-phase endometria were normalized to GAPDH expression, and the data for all of the endometrial samples are shown in the scatter plots. ^***^P < 0.001 compared with the proliferative endometrium. **c**, **d** Immunohistochemical analysis was performed using KLF12 antibody. Proliferative- and secretory-phase endometrial tissue samples from normal women are shown at ×200 (left panel) and ×400 (right panel) magnification. The negative control (NC) is nonspecific rabbit serum. Red represents positive staining (arrows). Scale bars, 100 μm (up panel) and 50 μm (below panel). The means and integrated optical densities of KLF12 expression in the endometrial tissue were calculated. **e** The Ishikawa cells were treated with estrogen (E_2_) (10^–8^ M) and progesterone (MPA) (10^–6^ M) at different times (process time: 0–72 h), as indicated. Whole-cell lysates were analyzed by western blot with the indicated antibodies. **f** Ishikawa cells were pretreated with ICI182780 and mifepristone for 12 h and then stimulated with E_2_ + MPA for another 12 h. KLF12 protein expression of these Ishikawa cells was determined by western blot. **g** Ishikawa cells were treated with multiple specific signaling pathway inhibitors for 1 h prior to 12 h treatment with E_2_ + MPA. The KLF12 expression level was determined by western blot analysis

The protein expression levels of KLF12 were lower in the secretory phase (*n* = 7) than in the proliferative phase of the endometrium (*n* = 6) (Fig. 3a, b). In addition, as demonstrated in the immunolocalization analysis, the KLF12 protein abundance was lower in the secretory phase than in the proliferative phase of the endometrium, especially in the endometrial epithelial cells (Fig. 3c, d). As shown in Fig. 3e, KLF12 protein expression decreased significantly and reached a minimum (~20% relative to 0 h) at 72 h after treatment with E_2_ and MPA. To determine the exact upstream sex hormone modulator of KLF12, KLF12 protein expression was examined in Ishikawa cells pretreated with ICI182780 and mifepristone before E_2_ or MPA treatment. As KLF12 protein expression reduced obviously at 12 h after E_2_ + MPA treatment (Fig. 3e), 12 h was chosen as the stimulation time for sex hormone. The results showed that the KLF12 protein level was reversed with the pretreatment of mifepristone performed before E_2_ plus MPA treatment, which indicated that KLF12 was a progestogen target gene (Fig. 3f). To further determine the molecular signaling pathways involved in the MPA-reduced expression of KLF12, Ishikawa cells were pretreated with various signaling pathway inhibitors for 1 h prior to MPA stimulation. As shown in Fig. 3g, MPA repressed KLF12 expression, and KLF12 expression was markedly reversed by the phosphatidylinositol-3 kinase (PI3K)/AKT pathway inhibitor LY294002. These results suggested that this pathway was involved in the repression of KLF12 expression during MPA stimulation in Ishikawa cells.

### KLF12 represses LIF expression in Ishikawa cells

**Fig. 4:**
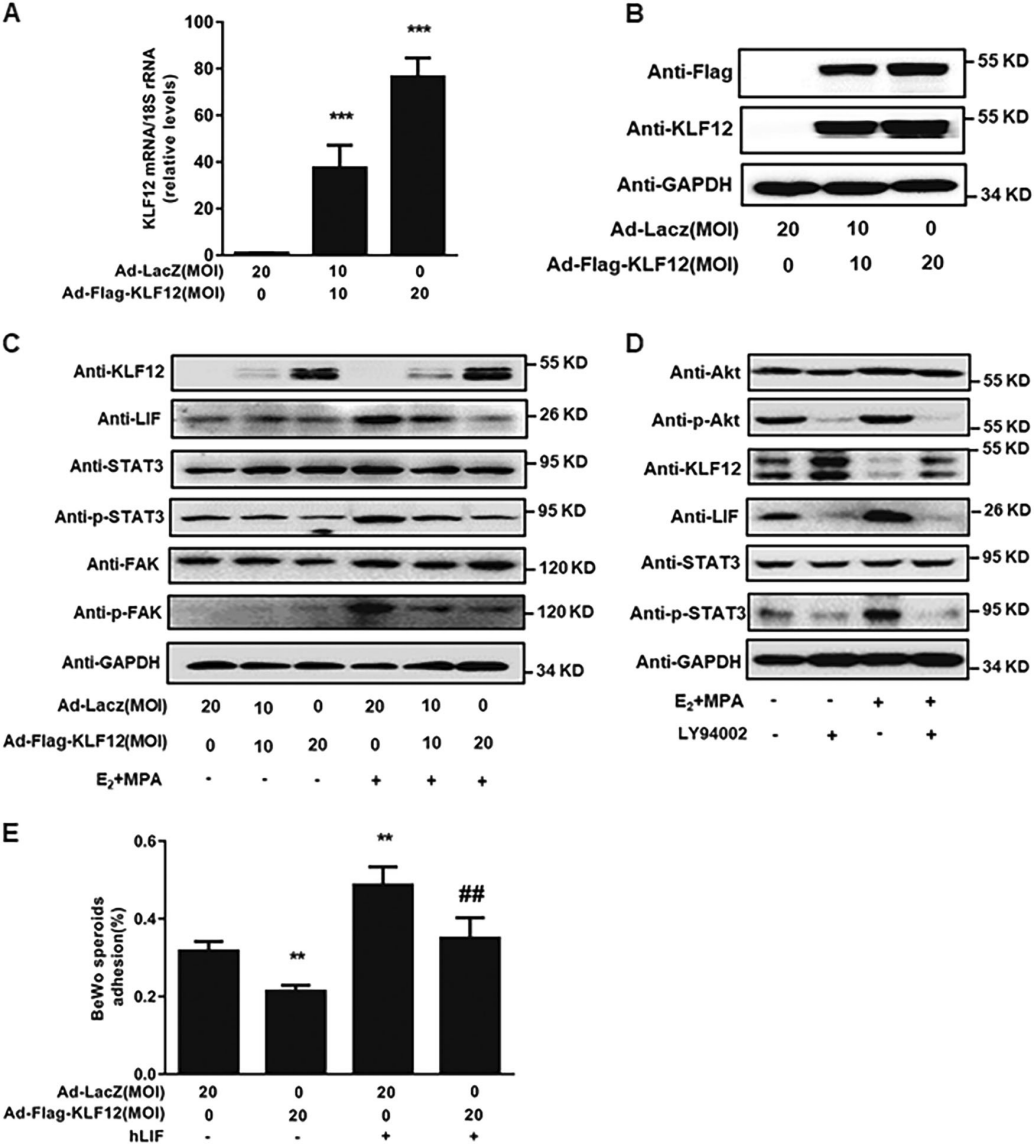
KLF12 represses LIF expression in Ishikawa cells. **a**, **b** Ishikawa cells were infected with an adenovirus expressing either LacZ or KLF12 for 48 h at the indicated MOI. The KLF12 transcript copy number and protein expression were determined by real-time PCR and western blotting, respectively. ^***^P < 0.001 compared with the Ad-LacZ only treatment group. **c** Ishikawa cells were infected with Ad-LacZ or Ad-Flag-KLF12 at the indicated MOI for 24 h and then treated with E_2_ and MPA for another 12 h; subsequently, the protein levels of LIF and its downstream targets, p-STAT3/STAT3 and p-FAK/FAK, were measured by western blot. **d** Ishikawa cells were treated with specific AKT pathway inhibitor for 1 h prior to 12 h treatment with E_2_ + MPA. The KLF12, LIF, and p-STAT3/STAT3 expression levels were determined by western blot analysis. **e** Ad-Flag-KLF12-treated cells were cultured with human recombinant LIF before the transfer of the BeWo spheroids. ^**^P < 0.01 compared with the Ad-LacZ group; ^##^*P* < 0.01 compared with the Ad-Flag-KLF12 group

As observed in Fig. 4c, KLF12 overexpression in Ishikawa cells (Fig. 4a, b) resulted in significant dose-dependent decreases in LIF protein abundance. In addition, the phosphorylation level of STAT3 was examined by western blot to confirm the biological function of LIF in embryo attachment. The phosphorylation level of STAT3 was reduced significantly in KLF12-overexpressing Ishikawa cells with E_2_ and MPA treatment (Fig. 4c). At the same time, some other signals, such as p-FAK/FAK, associated with embryo attachment, were decreased in Ishikawa cells infected with Ad-Flag-KLF12 with or without E_2_ and MPA stimulation (Fig. 4c). Furthermore, the results demonstrated that LY294002 could reduce the expression levels of LIF and p-STAT3 which were induced by E_2_ and MPA (Fig. 4d). Importantly, treating KLF12-enhanced Ishikawa cells with human recombinant LIF before BeWo spheroid transplantation onto these cells resulted in an improved adhesion rate, which indicated that LIF could rescue the KLF12-induced dysfunction of embryo adhesion (Fig. 4e).

### LIF is a novel target gene of KLF12 in Ishikawa cells

**Fig. 5:**
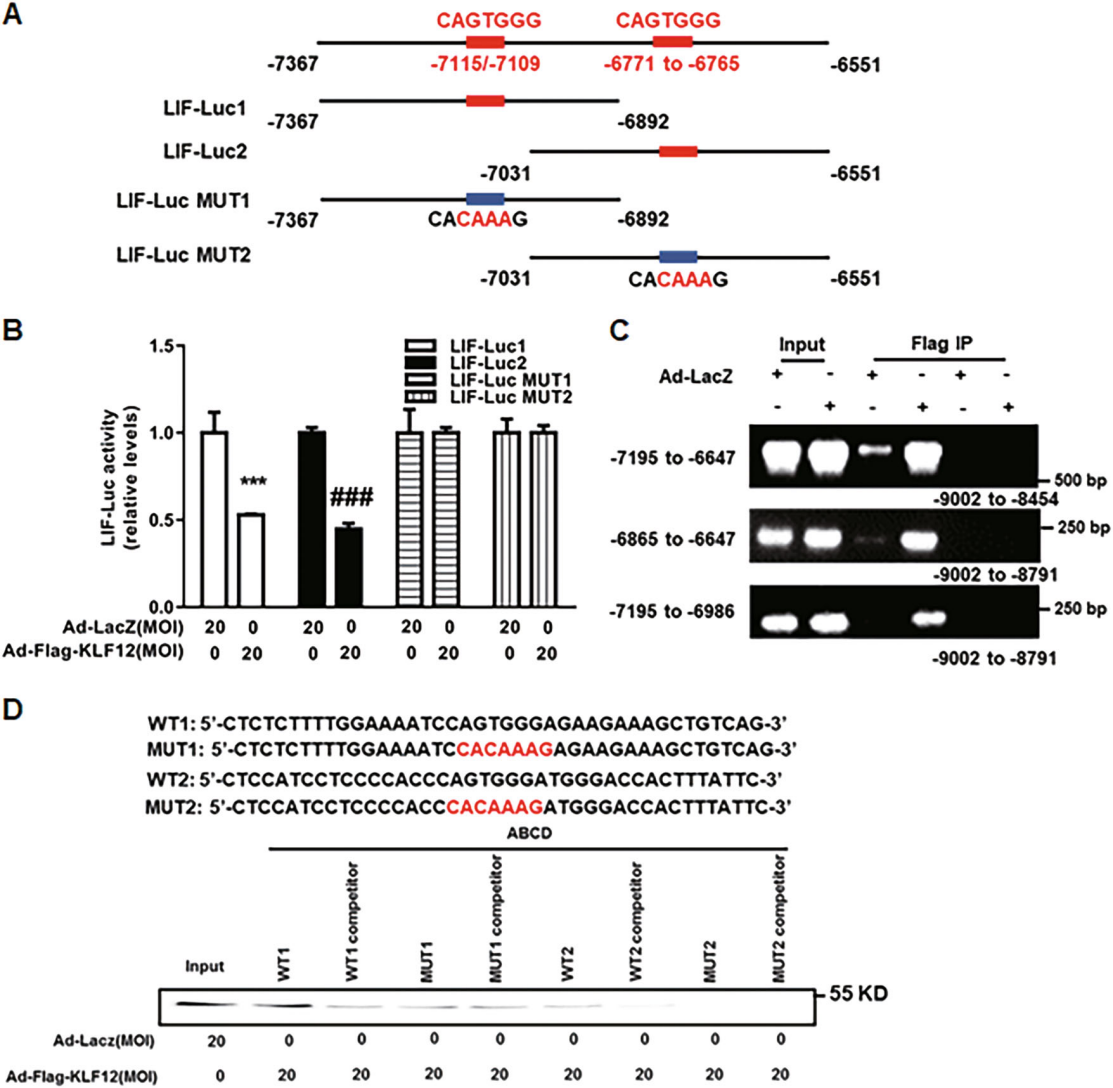
KLF12 directly repressed LIF transcription in Ishikawa cells. **a** Four LIF-Luc reporter constructs were designed according to the schematic diagram of partial LIF promoter sequence. These constructs were named as LIF-Luc1/2 and LIF-Luc Mut1/2. **b** Ishikawa cells were infected with the indicated adenoviruses for 24 h and then transfected with LIF-Luc1/2 or LIF-Luc MUT1/2. After 48 h, luciferase assays were performed, and the data were plotted after normalization to Renilla luciferase activity. ^***^P < 0.001 and ^###^*P* < 0.001 compared with Ad-LacZ alone. **c** ChIP-PCR amplification using primers against the human LIF promoter region. PCR products were separated by agarose gel electrophoresis. Input (non-precipitated) chromatin was utilized as a positive control in these analyses. **d** ABCD assays were performed using biotinylated or non-biotinylated (competitor) double-stranded LIF wild-type (WT) and conserved element-mutated (MUT) oligonucleotides with whole-cell extracts from Ishikawa cells

Based on the human LIF promoter sequence, we found two conserved KLF12-binding sites (CAGTGGG) within the core region (−8000 to +100) of the LIF promoter. To localize the KLF12-binding site within this promoter, we generated 4 LIF-Luc reporter constructs. These constructs, namely, LIF-Luc1/2 and LIF-Luc Mut1/2 (Fig. 5a), were used in luciferase reporter assays. The luciferase reporter assay results demonstrated that LIF promoter activity could be repressed by KLF12 overexpression in Ishikawa cells, while the mutational LIF promoter activity remained unchanged (Fig. 5b). Next, conventional ChIP-PCR analysis was further conducted to investigate whether the LIF promoter was a direct target of KLF12 in Ishikawa cells. As shown in Fig. 5c, two promoter regions (−6865 to −6647 bp and −7195 to −6986 bp) were effectively recovered from immunoprecipitates of the Flag-KLF12 protein, but they were not recovered from those of the LacZ control. In addition, no PCR product was obtained from the Flag-KLF12 or LacZ control immunoprecipitate using the negative control primers (−9002 to −8791 bp, Fig. 5c). Furthermore, we performed ABCD assays using biotinylated double-stranded oligonucleotides WT1 (−6790/−6749), WT2 (−7131/−7091), and MUT1/MUT2, which were derived from the LIF promoter sequence. We observed that the Flag-tagged KLF12 protein strongly bound to WT1 and WT2 but not to MUT1 or MUT2.

### Reduction in LIF expression in the endometria of RIF patients

**Fig. 6:**
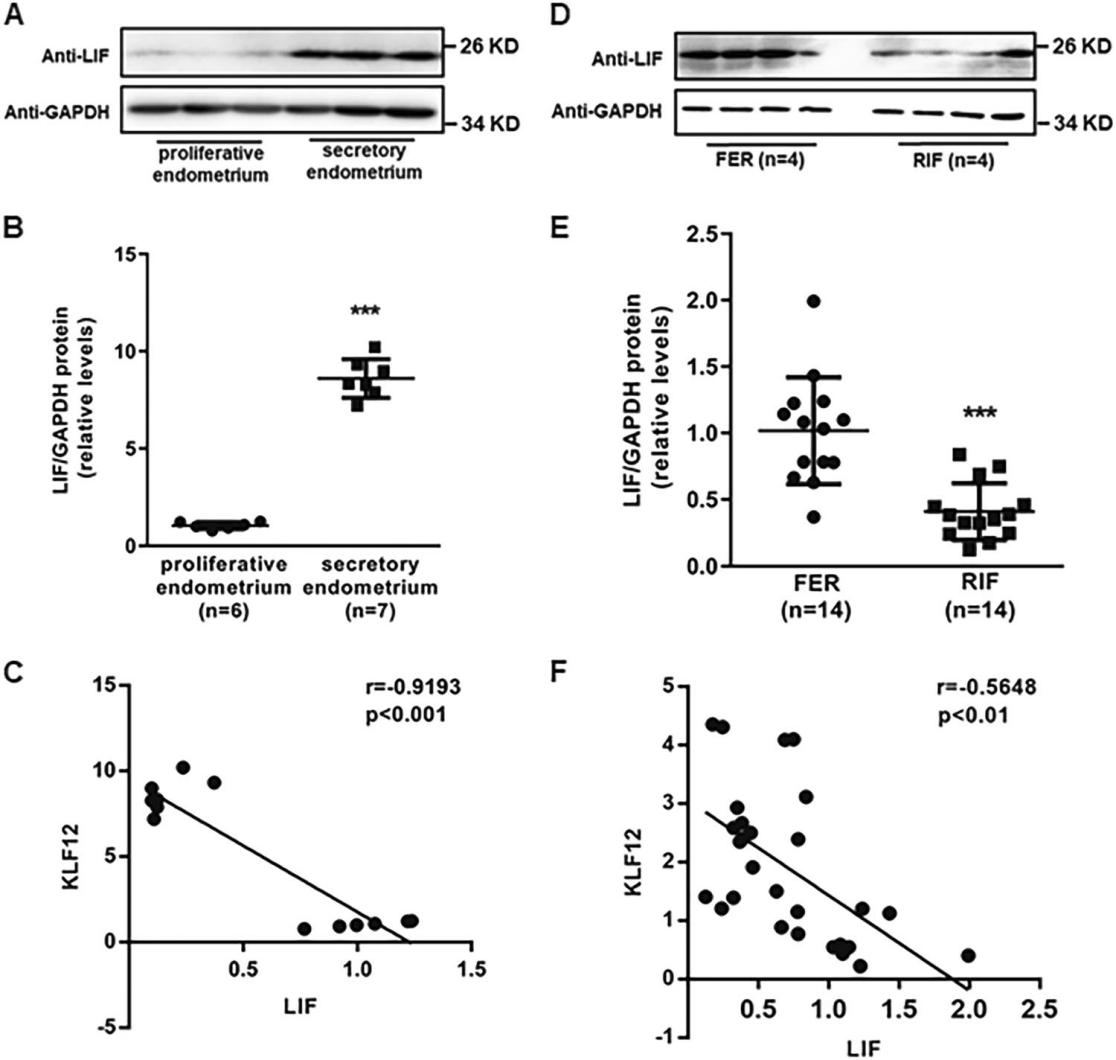
Correlation of KLF12 and LIF in the endometrium. **a**, **b** The total LIF protein levels of the FER and RIF endometria were normalized to GAPDH expression, and the data for all of the endometrial samples are shown in the scatter plots. ^***^P < 0.001 compared with the FER control. **c** Correlation between KLF12 and LIF protein expression in the proliferative- and secretory-phase endometria (*n* = 13, *r* = −0.9193, *P* < 0.001). **d**, **e** The total LIF protein levels of the proliferative- and secretory-phase endometrium were normalized to GAPDH expression, and the data for all of the endometrial samples are shown in the scatter plots. ^***^P < 0.001 compared with the proliferative endometrium. **f** Correlation between KLF12 and LIF protein expression in the RIF and fertile control endometria (*n* = 28, *r* = −0.5648, *P* < 0.01)

To examine the correlation between KLF12 and LIF in the human endometrium, we examined LIF protein expression in the proliferative phase and secretory phase of the endometria of normal cycling women. The protein expression levels of LIF were higher in the secretory-phase endometrium (*n* = 8) than in the proliferative-phase endometrium (*n* = 8) (Fig. 6a, b). Furthermore, as shown in Fig. 6c, the protein levels of these two factors were highly negatively correlated (*r* = −0.9193, *P* < 0.001) in these endometrial samples. As KLF12 has been proved to be decreased in the endometria of RIF patients, we investigated LIF expression in endometrial samples from RIF and fertile women. As expected, LIF expression was significantly reduced by 50% in the endometria of the RIF patients compared with the endometria of the fertile controls (Fig. 6d, e). In addition, the protein levels of these two factors were moderately negatively correlated (*r* = −0.5648, *P* < 0.01) in both the RIF and control endometria (Fig. 6f).

## Discussion

Implantation is a complex initial step in the establishment of a successful pregnancy, and a receptive endometrium is required for the initiation of the maternal–fetal interaction. The localization and adhesion of the embryo to the epithelial cells is the first step in the initiation of implantation. Suboptimal embryo adhesion is widely thought to be a major cause of implantation failure^25,26^. Studies have shown that many factors are involved in regulating embryo adhesion processes. In this study, we revealed that KLF12 impaired embryo adhesion by repressing LIF expression.

A receptive uterus for embryo adhesion is defined as a spatial- and temporal-specific period that is regulated by maternal estrogen and progesterone^27^. LIF, a pro-inflammatory cytokine, plays a pivotal role in regulating embryo attachment^28^. Embryos from mice that are null for *Lif* can develop normally, but their implantation is blocked. *Lif* knockout mouse embryos can implant normally when transferred to wild-type pseudo-pregnant females, indicating that a maternal defect is responsible for the implantation failure^12,15^. The addition of exogenous LIF to 3.5 dpc or 4.5 dpc mice that are null for *Lif* can rescue the impaired embryo implantation in *Lif* or *p53* knockout mice^12,29^. LIF is an essential regulator of embryo implantation in mice and is thought to be required in several mammalian species, including humans. Deregulated LIF expression is linked to several cases of female infertility that are associated with defective endometrial receptivity. Studies have reported that the expression of LIF in the endometrial tissue of infertile women is closely related to the clinical pregnancy rate of the IVF-ET cycle^30^. Our study together with other studies confirms that LIF is significantly decreased in endometria from women with infertility-causing conditions, such as RIF^30,31^. However, subcutaneous injection of exogenous hLIF fails to improve the pregnancy outcomes of RIF patients^32^. To date, the specific mechanisms of LIF expression and regulation have remained unclear. Progesterone and certain cytokines (such as HB-EGF and TGF-β1) can reportedly regulate the expression of LIF in endometrial epithelial cells^33,34^. The expression of *Lif* in endometrial epithelial cells of Foxa2, Hoxa11, and P53 knockout mice is also significantly reduced^35,36^. Moreover, the embryo adhesion process is impaired, and the expression of endometrial KLF12 protein is increased in RIF patients. Further experiments were employed to reveal the exact relationship between KLF12 and LIF. As a conserved KLF12-binding sequence, CAGTGGG was found in the promoter region of LIF, and ChIP/PCR and ABCD assays were applied to demonstrate that KLF12 could specifically bind to the LIF promoter and repress LIF gene expression. Furthermore, KLF12 repressed embryo attachment by decreasing LIF expression. In this study, we have comprehensively examined the effect of KLF12 on embryo adhesion in RIF patients.

On the other hand, after the blastocyst becomes juxtaposed and adheres to the luminal epithelium, the embryo invades into the endometrial stroma^5,6^. The adequate decidualization of hESCs, which is defined by the mesenchymal-to-epithelial transformation of endometrial fibroblasts and stromal cells into secretory epithelioid decidual cells, is a precondition for embryo invasion^37^. Our previous study demonstrated that increased endometrial KLF12 could disrupt decidualization by repressing Nur77 expression. LIF is also an important regulator of decidualization as the uteri of *Lif* ^−/−^ mice do not decidualize, even in response to different well-established stimuli that induce this process^38^. LIF induces the β-catenin pathway by activating the ligand Wnt7A specifically in the subepithelial stroma, which has important roles in the decidual process^39,40^. LIF also regulates other signaling in LE, affecting decidualization via changes in the expression of several ligands and receptors, such as *Msh* homeobox 1 (MSX1)^40–42^, *Ror2* receptor and Frizzled 6. Hence, the dysfunction of KLF12-decreased LIF expression in decidualization in RIF patients should be further investigated, and the mechanism of the intercellular interactions between epithelial cells and stroma cells in RIF patients deserves further attention.

KLF12, a member of the KLF family of zinc-finger transcription factors, is a critical regulator of cell differentiation, phenotypic modulation, and physiological function^43^. The KLF family plays multiple roles in embryo implantation in vitro and in vivo. For example, KLF5 is critical for decidualization, and mice that are null for KLF5 have evident subfertility^44^. In addition, KLF15 is a hormone-related gene that blocks Ishikawa cell proliferation by binding to the Mcm2 promoter^45^. Furthermore, KLF12, a transcription factor that binds to the promoter regions of target genes and represses their expression through an N-terminal PVDLS sequence (Pro-Xaa-Asp-Leu-Ser), recognizes and interacts with the CAGTGGG sequence^46,47^. KLF12 can interrupt embryo implantation, which is due to the endometrial receptive dysfunction of RIF patients. As embryo implantation is a process that is regulated by ovarian estrogen and progestogen, LIF is maximally expressed in the endometrial GE, just prior to implantation^15^. At the same time, KLF12 is considered an implantation-associated gene that is regulated by estrogen and progestogen in our study. An ER inhibitor and PR inhibitor were both used to dissect the regulatory mechanism of disorganized KLF12 expression. KLF12 was shown to be the downstream gene of progestogen. Activation of the PI3K/AKT signaling pathway plays an important role in embryo adhesion^48^. The phosphorylation of AKT can affect hESC decidualization in endometriosis and adenomyosis^49–51^. We speculated that the dysregulation of several pathways under estrogen and progestogen led to enhanced KLF12 in RIF patients. In the current study, we found that the PI3K/AKT signaling pathway, and not the PKA, MEK1/2 or p38 MAPK signaling pathways, induced KLF12 expression, which was reduced by estrogen and progestogen. This suggests that the signaling cascade contributing to embryo adhesion might be impaired in women with RIF.

The activation of the LIF-regulated JAK–STAT3 pathway, specifically in the LE, has various functions in embryo implantation. We have observed decreased p-STAT3/STAT3 expression in KLF12-enhanced Ishikawa cells. In addition, some other pathways activated by LIF are also already established as essential for attaining embryo implantation. These pathways regulate various functions in the uterus. Pathways such as VEGF/HIF-1a, FGF, and NOTCH regulate vasculogenesis, while the TOLL-NF-kB pathways are involved in inflammation, innate immunity, and cell stress/apoptosis. The canonical pathways induced by mTOR, IGF1, and the TGFb superfamilies and FGF pathways regulate metabolism and cell proliferation within the different uterine compartments, while the actin cytoskeleton and WNT and ephrin pathways may regulate cytoskeletal organization/cell adhesion in the LE^41^. These pathways mediate changes in uterine physiology, including epithelial proliferation, polarity, angiogenesis, innate immune response, and stromal cell decidualization. We plan to investigate more functions of the KLF12-LIF interaction in embryo implantation. A new mechanism of impaired endometrial receptivity in RIF patients will be revealed and will be critical for the improved treatment of patients with conditions of insufficient endometrial receptivity-associated infertility.

This study is the first to functionally analyze the roles of KLF12 and LIF in poor endometrial receptivity. KLF12 is pathologically enhanced partly due to dysregulation of ovarian sex hormones in the endometria of RIF patients. In addition, our results have revealed a new potential mechanism of impaired embryo adhesion that may be relevant in patients with implantation failure, which will help to resolve endometrial capacity-relevant infertility conditions in IVF programs.

## Materials and methods

### Patient samples

The patients involved in this study were recruited from the IVF unit of the Reproductive Center of the Affiliated Drum Tower Hospital of Nanjing University Medical School from January 2016 to August 2016. A total of 17 RIF patients, who had failed to achieve pregnancy after at least four good-quality embryo transfers over a minimum of three fresh or frozen cycles, were enrolled. A total of 6 proliferative-phase endometria were obtained from patients undergoing endometrial biopsy within days 5–12 of menstruation. Seven secretory-phase endometrial tissues were obtained from age-matched fertile controls within days 19–23 of menstruation via diagnostic uterine curettage. The use of human tissues was approved by the institutional review board of the Drum Tower Hospital of Nanjing University on 5 December 2013 (2013-081-01). Women with polycystic ovarian syndrome (PCOS), hydrosalpinx, endometriosis, adenomyosis, endometrial hyperplasia or endometrial polyps were excluded. The details of these patients are listed in Tables 1 and  2.

**Table. 1:** Demographic details of the participants (FER&RIF) in this study

Fertile	FER (*n* = 17)	RIF (*n* = 17)	*P*
Age (years)	30.1 ± 4.3	30.3 ± 2.1	ns
Body mass index (kg/m^2^)	21.7 ± 2.7	21.9 ± 2.5	ns
Menstrual cycle (days)	28.1 ± 2.2	30.2 ± 2.7	ns
Endometrial thickness (mm)	10.8 ± 1.7	9.6 ± 1.5	ns
No. of transferred embryos	2 ± 0	8.4 ± 4.2	s

The data are presented as the mean ± SD unless otherwise indicated

**Table. 2:** Demographic details of the participants (proliferative and secretory phase) in this study

Fertile	Proliferative phase (*n* = 6)	Secretory phase (*n* = 7)	*P*
Age (years)	33.5 ± 2.7	34.4 ± 3.5	ns
Body mass index (kg/m^2^)	20.9 ± 2.2	21.3 ± 2.6	ns

The data are presented as the mean ± SD unless otherwise indicated

### Cell culture

The Ishikawa cells and BeWo cells used in this study were donated by Dr. Yali Hu from Department of Gynecology and Obstetrics in the Affiliated Drum Tower Hospital of Nanjing University Medical School. The cells were cultured in Dulbecco’s modified minimum essential medium F12 (HyClone, Thermo Scientific, South Logan, UT, USA) containing 10% fetal bovine serum (FBS, Gibco BRL/Invitrogen, Carlsbad, CA, USA) and 1% penicillin/streptomycin (HyClone, Thermo Scientific, South Logan, UT, USA). Ishikawa cells were cultured for the indicated durations under the treatments described in each figure legend. Treatments included 10 nM E_2_ and 1 μM MPA (Sigma, St. Louis, MO, USA) for different times (Fig. 3e: 0–72 h; Other Figures: 12 h) with 2% charcoal/dextran-treated FBS and 1% penicillin/streptomycin in DMEM/F12 at 37 °C. ICI182780 (V900926, Sigma, St. Louis, MO, USA) and mifepristone (M8046, Sigma, St. Louis, MO, USA) were used at a concentration of 1 μM for 24 h to block estrogen receptors (ESRs) and progesterone receptors (PRs) in Ishikawa cells.

### Construction of adenoviruses

Adenoviruses harboring full-length KLF12 (Ad-Flag-KLF12, NCBI Reference Sequence: NM_007249.4) were generated using an AdMax (Microbix Biosystems, Inc., Toronto, Canada) system, according to the manufacturer’s recommendations. The viruses were packaged and amplified in HEK293A cells and purified by CsCl banding.

### RNA isolation and quantitative real-time PCR (qRT-PCR)

Total RNA was extracted from Ishikawa cells using the Trizol reagent (Invitrogen, Carlsbad, CA, USA), according to the manufacturer’s instructions. A total of 1 μg of total RNA was reverse transcribed into cDNA using a Prime Script RT reagent kit (Takara-Bio, Otsu, Japan). Reverse transcription was performed using random primers, and qRT-PCR was conducted with a MyiQ Single-Color Real-Time PCR Detection System (Bio-Rad, Hercules, CA, USA). The following primers were also used for the indicated genes: KLF12, 5′-CCTTTCCATAGCCAGAGCAG-3′ and 5′-TTGCATCCCTCAAAATCACA-3′; LIF, 5′-AGTCGTGACCTTGGCACCTC-3′ and 5′-GTTGACAGCCCAGCTTCTTC-3′; and 18S rRNA, 5′-CGGCTACCACATCCAAGGAA-3′ and 5′-CTGGAATTACCGCGGCT-3′. The PCR conditions were as follows: 95 °C for 15 min, followed by 40 cycles of 95 °C for 15 s and 60 °C for 30 s. Reactions were run in duplicate using RNA samples from three independent experiments. The fold change in the expression of each gene was calculated using the 2^−△△CT^ method, in which genes expression was normalized to the expression of the endogenous control (18S rRNA).

### Mouse experiments

ICR mice were purchased from the Laboratory Animal Center of Yangzhou University (Yangzhou, China) and were bred at the Laboratory Animal Center of Nanjing Drum Tower Hospital (Nanjing, China). All studies were approved by the Institutional Animal Care and Use Committee of Nanjing Drum Tower Hospital (SYXK 2014–0052). Females (6 weeks old) were mated with fertile males, and the morning on which a vaginal plug was observed was considered as day 0.5 of pregnancy. Twenty microliters (2 × 10^8^ TU/side) of Ad-LacZ or Ad-Flag-KLF12 was injected into the bilateral uterine horns of the mice in the morning on 1.5 dpc. On 4.5 dpc, the implantation sites were visualized after an intravenous injection of Chicago blue dye. Non-implanted blastocysts were flushed out of the uterus, and the representative image is shown.

### Western blotting

Tissues and cells were homogenized in whole-cell lysis buffer (50 mM Tris HCl (pH 7.6), 150 mM NaCl and 1.0% NP-40) containing a protease inhibitor cocktail and phosphatase inhibitor cocktails 2 and 3 (Sigma, St. Louis, MO, USA). Equal amounts of total protein (25–60 µg) were separated on a 10–15% (w/v) SDS-polyacrylamide gel and transferred onto polyvinylidene fluoride membranes (Millipore, Billerica, MA, USA). The membranes were blocked in Tris-buffered saline solution containing 5% nonfat milk for 1 h, and immunoblotting was performed with a primary antibody against KLF12 (1:2000; sc-84347, rabbit polyclonal antibody, Santa Cruz Biotechnology, Santa Cruz), LIF (1:250; ab113262, rabbit polyclonal antibody, Abcam), STAT3 (1:1000; 4904, rabbit monoclonal antibody, CST), p(Y705)-STAT3 (1:250; AP0247, rabbit polyclonal antibody, Bioworld Technology), FAK (1:1000, BS3581, rabbit polyclonal antibody, Bioworld Technology), p-FAK(Y925) (1:1000, BS4718, rabbit polyclonal antibody, Bioworld Technology), AKT (1:1000; Bioworld Technology), or p(Ser473)-AKT (1:1000; Santa Cruz Biotechnology), followed by incubations with goat anti-rabbit or rabbit anti-mouse HRP-conjugated secondary antibodies. Detection was performed using an enhanced chemiluminescence kit (Millipore). GAPDH (1:10,000; AP0063, GAPDH polyclonal antibody, Bioworld Technology) served as the internal control to indicate the total protein levels in lysates.

### Immunohistochemical staining

After the endometrial samples were dewaxed, endogenous peroxidase activity was blocked using freshly prepared PBS containing 0.3% hydrogen peroxide for 10 min. Antigen retrieval was conducted by autoclaving the samples at 121 °C for 15 min in the presence of EDTA (pH = 9.0), followed by incubation in blocking solution to block endogenous biotin for 30 min. The sections were washed with TBS and then incubated with antibodies against KLF12 (1:600; sc-84347, rabbit polyclonal antibody, Santa Cruz Biotechnology, Santa Cruz, CA, USA) overnight at 4 °C in a humidified chamber. Subsequently, the sections were rinsed with PBS and incubated with an HRP-conjugated goat anti-rabbit secondary antibody at 37 °C for 20 min. HRP activity was detected using diaminobenzidine (Invitrogen, Carlsbad, CA, USA), and the sections were counterstained with hematoxylin. Control sections were run concurrently with the experimental sections using nonspecific rabbit IgG, and these sections were similarly pretreated. Nonspecific staining was not detected in the controls.

### Attachment assay for BeWo spheroid and mouse embryo attachment to Ishikawa cells

Ishikawa cells were seeded into a 24-well plate and transfected with the indicated adenovirus. Then, BeWo cells were detached with 0.25% trypsin (Gibco BRL/Invitrogen, Carlsbad, CA, USA) after reaching 80% confluence. The BeWo cell suspensions were placed in the 35-mm^2^ dishes coated with an anti-adhesive polymer, poly-2-hydroxyethyl methacrylate (Sigma, St. Louis, MO, USA), to induce the formation of BeWo spheroids that were 150–200 μm in diameter after 48 h of culture. The spheroids were then transferred onto a confluent monolayer of Ishikawa cells. After incubation at 37 °C for 2 h, nonadherent spheroids were removed by washing with PBS containing Ca^2+^ (0.1 mg/L) and Mg^2+^ (0.1 mg/L). The attached spheroids were counted, and the attachment rate was expressed as a percentage of the total number of spheroids added to the Ishikawa monolayer (% adhesion). All cocultures were monitored using a fluorescence microscope (Leica).

Similarly, collected mouse blastocysts were transferred onto Ishikawa cells infected with the indicated adenovirus vectors in a 24-well culture plate, with 10 blastocysts per well. A standardized plate movement protocol was implemented to measure the embryo attachment stability after 24 h.

### Luciferase reporter assay

The pGL3-basic luciferase reporter plasmids loaded with the LIF promoter (Luc1/Luc2/Luc MUT1/Luc MUT2) were employed in this experiment. Pre-confluent (70%) Ishikawa cells in 12-well plates were transfected with the indicated plasmids using the MaxPEI transfection reagent (TG1002, Tigergene Technologies, PA, USA). Cells were harvested, and the luciferase activities were analyzed after 48 h using the Dual-Luciferase Assay System (Promega, Madison, WI, USA). Luciferase activity was measured using a luminescence counter (Berthold Technologies, KG, Germany) according to the manufacturer’s instructions. To assess transfection efficiency, firefly luciferase activity was normalized to the corresponding Renilla luciferase activity.

### Chromatin immunoprecipitation (ChIP)/PCR assay

Ishikawa cells (70% confluence) were infected with Ad-LacZ and Ad-Flag-KLF12 (at a multiplicity of infection (MOI) of 20) for 48 h. Next, the treated cells were prepared for ChIP using Flag beads as described previously^52^. The recovered DNA was analyzed by RT-PCR. The PCR mixtures contained 2 μL of DNA, standard PCR reagents, and 50 pmol of each primer (LIF P1: 5′-TCCAGGAAATCATTGAGTGACA-3′ and 5′-CCAGCAGCTGGCTTCCTCCC-3′ and P2: 5′-GGAAGCTCACTCTTCCTTCT-3′ and 5′-CACCCTGACCCGACCCAGCA-3′, specific for LIF promoter DNA fragments). Negative control primers were set to target distal sequence (−9002 to −8791 bp and −9002 to 8454 bp).

### Avidin–biotin conjugate DNA precipitation assay

The following double-stranded oligonucleotides were used for the avidin–biotin conjugate DNA precipitation (ABCD) assay: LIF WT1: 5′-CTCTCTTTTGGAAAATCCAGTGGGAGAAGAAAGCTGTCAG-3′; LIF WT2: 5′-CTCCATCCTCCCCACCCAGTGGGATGGGACCACTTTATTC-3′; LIF MUT1: 5′-CTCTCTTTTGGAAAATCCACAAAGAGAAGAAAGCTGTCAG-3′ and LIF MUT2: 5′-CTCCATCCTCCCCACCCACAAAGATGGGACCACTTTATTC-3′. The primers were biotinylated at the 5-end of the sense strand. Ishikawa cells were infected with Ad-LacZ and Ad-Flag-KLF12 (MOI = 20) for 48 h. Cell extracts were harvested and lysed in RIPA buffer. The ABCD assay was performed by incubating 1000 μg of precleared cell extracts derived from Ishikawa cells with 500 pmol of double-stranded DNA immobilized on streptavidin agarose in binding buffer (10 mM Tris, pH 8.0, 150 mM NaCl, 0.5% (v/v) Triton X-100, 0.5 mM DTT, 0.5 mM EDTA, 10% (v/v) glycerol, 20 μg/ml poly [dI-dC], and protease inhibitor cocktail). After incubation for 4 h at 4 °C, the beads were washed four times with the same buffer. Proteins were resolved by SDS-PAGE, followed by electrotransfer onto a polyvinylidene fluoride membrane. Probing was performed with a Flag-HRP antibody (1:5000; A8592, Sigma, St. Louis, MO, USA). Immunodetection was accomplished by enhanced chemiluminescence (Millipore, Billerica, MA, USA).

### Statistical analysis

The data are presented as the mean ± SEM. All experiments were performed at least three times. Student’s t-test was used for comparisons between two groups. Statistical analysis was conducted by ANOVA, followed by the Student–Newman–Keuls test, for experiments involving more than two groups. Pearson correlation analysis was used to assess the relationship between KLF12 and LIF. *P* values <0.05 were considered statistically significant.
